# Optimizing Broiler Diets: Nutritional and Functional Benefits of Fermented Cottonseed Meal

**DOI:** 10.1002/fsn3.71765

**Published:** 2026-07-03

**Authors:** Dan Song, Qiuxi Xuan, Beibei He, Xiaolin Hou, Yongwei Wang, Weiwei Wang, Kuanbo Liu, Tao Duan, Junlin Cheng, Aike Li, Lin Qiao

**Affiliations:** ^1^ Key Laboratory of Grain and Oil Biotechnology Academy of National Food and Strategic Reserves Administration Beijing P.R. China; ^2^ Animal Science and Technology College Beijing University of Agriculture Beijing P.R. China

**Keywords:** broiler, fermented cottonseed meal, growth performance, intestinal morphology, short‐chain fatty acid

## Abstract

Cottonseed meal (CSM) contains antinutritional factors limiting its use. Fermentation may improve its nutritional value. This study examined the nutritional properties of fermented cottonseed meal (FCSM) and its effects on broilers. The nutritional composition of fermented CSM and FCSM was analyzed. Subsequently, 175 AA broilers were randomly assigned to five dietary treatments: a basal control diet (CON), and diets containing 6% CSM, 6% FCSM, 9% FCSM, or 12% FCSM. Fermentation significantly increased crude protein, acid‐soluble protein, *L*‐lactic acid, and gross energy of CSM (*p* < 0.05), while decreasing free gossypol and crude fiber content (*p* < 0.05). FCSM also significantly enhanced the standardized ileal digestibility of glutamate and valine (*p* < 0.05), but not other amino acids. In broilers, dietary inclusion of 9% FCSM increased body weight and average daily gain during the starter phase compared to the 6% CSM diet (*p* < 0.05). At day 21, both 6% and 9% FCSM diets significantly increased serum concentrations of IgA, IgG, and IgM compared to the CON group (*p* < 0.05). The 6% FCSM group demonstrated a significant enhancement in the villus height to crypt depth ratio compared to the 6% CSM diet (*p* < 0.05). Additionally, 6% FCSM significantly raised cecal concentrations of acetate, propionate, butyrate, and total short‐chain fatty acids at 21 days (*p* < 0.05). In conclusion, fermentation enhances the nutritional profile of CSM by improving protein utilization and reducing antinutritional factors. Dietary inclusion of 6%–9% FCSM improves starter‐phase growth performance, immune function, and intestinal health in broilers.

## Graphical abstract

Fermentation significantly improved the nutritional value of cottonseed meal (CSM) by increasing crude protein, acid‐soluble protein, *L*‐lactic acid, and gross energy, while reducing free gossypol and crude fiber. In broiler trials, dietary supplementation with fermented CSM (FCSM) significantly enhanced growth performance, increased serum immunoglobulins, improved intestinal villus structure, raised cecal SCFA concentrations and optimized the cecal microbial community structure. Diets containing 6%–9% FCSM are a beneficial feed ingredient that supports growth, immunity, and intestinal health in broilers.

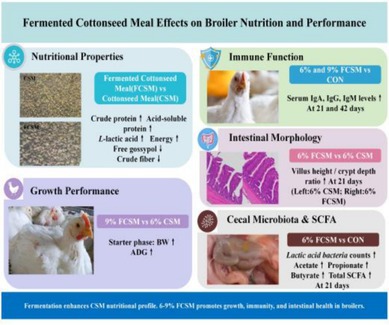

## 1 Introduction

The continuous growth of the global population is driving an escalating demand for animal‐derived protein (Weimer et al. 2022). Poultry meat, in particular, has witnessed a substantial increase in consumption due to its high production efficiency and relatively low environmental impact (Liu et al. 2020). According to the 2023 report by the Food and Agriculture Organization of the United Nations (FAO), global poultry meat production has surpassed 126 million tons, with China contributing 12.23% to the total output (FAOSTAT 2025). In response to the continuously increasing demand for meat, it is imperative to make livestock production more efficient and sustainable. Soybean meal (SBM) is extensively used as a primary protein source in poultry diets due to its high crude protein (CP) content and widespread availability (Ashayerizadeh, Jazi, Rezvani, et al. 2024). However, its application is increasingly challenged by market price volatility and supply chain instability (Munawar et al. 2025). Consequently, the development of economically viable and supply‐stable alternative protein sources has become a critical focus. Cottonseed meal (CSM), a major by‐product generated from the dehulling and pressing of cottonseeds, contains approximately 43% to 52% CP and is rich in amino acids and vitamins, demonstrating considerable potential as a plant‐based protein feed resource (Liu et al. 2024). As the world's second‐largest producer of cottonseed, China supplied more than 4000 t of cottonseed in 2020, thereby providing a reliable supply base for CSM (Yan et al. 2025). Despite being acknowledged as a high‐quality protein source, the utilization of CSM in animal feed is constrained by several antinutritional factors, such as free gossypol (a toxic polyphenolic pigment), cyclopropenoid fatty acids, elevated crude fiber (CF) content, and certain amino acid imbalances (Esen 2024). Among these factors, free gossypol is particularly toxic, potentially impairing animal development and causing visceral abnormalities (Rehemujiang et al. 2023). To enhance the nutritional value of CSM, various detoxification and nutritional improvement strategies have been developed, including physical and chemical treatments, as well as microbial fermentation (Liu et al. 2024). Microbial fermentation is regarded as a particularly effective biological processing strategy due to its ability to simultaneously reduce free gossypol content, improve protein digestibility, and enhance overall nutritional quality (Liu et al. 2024). Fermentation using *Bacillus subtilis* ST‐141 and *yeast* N5 has been shown to significantly reduce the free gossypol content in CSM from 820 to 346 mg/kg, representing a 57.8% reduction. This process also markedly increased serum IgM levels by 72.97%, enhanced cecal *Lactobacilli* counts, and improved antioxidant capacity in broilers, without adversely affecting their growth performance (Wang et al. 2017). Previous research has indicated that replacing 50% of SBM with CSM fermented by *Bacillus subtilis* PTCC1156 and *Lactiplantibacillus plantarum* PTCC1058 enhances growth performance, nutrient digestibility, and digestive enzyme activity, while also modulating gut microbiota and reducing ammonia emissions (Ashayerizadeh, Jazi, Sharifi, et al. 2024). However, significant variability in the nutritional quality of CSM fermented with different microbial strains poses a challenge to its widespread application (Xie et al. 2024). Current research has primarily concentrated on gossypol detoxification, yet a systematic evaluation of post‐fermentation amino acid digestibility remains insufficient. Furthermore, the effects of fermented cottonseed meal (FCSM) on intestinal microbial metabolites, particularly short‐chain fatty acids (SCFA) profiles, and its regulatory mechanisms linking to intestinal mucosal morphology in broilers are not well understood.

The previous studies by the authors have shown that FCSM with a mixture of *Bacillus subtilis* BS15‐3, *Saccharomyces cerevisiae* SC17‐1, and *Lactiplantibacillus plantarum* LP15‐1 can significantly reduce the free gossypol content of CSM, increase the acid‐soluble protein content, and promote the production of *L*‐lactic acid in CSM to improve its palatability. Among these strains, *Saccharomyces cerevisiae* SC17‐1 mainly functions to degrade free gossypol, *Lactiplantibacillus plantarum* LP15‐1 is primarily responsible for producing *L*‐lactic acid, and *Bacillus subtilis* BS15‐3 mainly serves to enhance the acid‐soluble protein content of CSM. Fermentation of CSM with the combination of these three strains can significantly reduce the toxicity of CSM and improve its nutritional value (Xuan et al. 2022). Therefore, this study aims to quantitatively assess the transformation of nutritional components during the solid‐state fermentation of CSM. It seeks to determine the optimal dietary inclusion level of FCSM in broiler diets without compromising feed efficiency, and to elucidate its effects on growth performance, immune function, cecal SCFA composition and intestinal health. These findings are expected to provide a theoretical foundation for the rational application of FCSM in poultry nutrition.

## 2 Materials and Methods

The animal experiments were approved by the animal ethics committee of the Academy of National Food and Strategic Reserves Administration (ANFSRA, Beijing, China; Approval No. 2021L30) and conducted in compliance with institutional ethical guidelines.

### 2.1 Preparation of FCSM

Strains of *Bacillus subtilis* BS15‐3, *Saccharomyces cerevisiae* SC17‐1, and *Lactiplantibacillus plantarum* LP15‐1 were preserved in the Food Quality and Nutrition Institute, ANFSRA (Beijing, China). As solid starter cultures, the initial viable bacterial counts were all 5.0 × 10^10^ CFU/g. These three solid starter cultures were pre‐cultured in sterile water with 2% molasses for 4 h on a rotary shaker at 37°C and 180 rpm for *Bacillus subtilis* BS15‐3, at 30°C and 180 rpm for *Saccharomyces cerevisiae* SC17‐1, and in a constant temperature incubator at 37°C for *Lactiplantibacillus plantarum* LP15‐1, respectively. In the solid‐state fermentation process of CSM, a substrate composed of 98% CSM and 2% molasses was combined with water at a ratio of 1:0.8 (w:w). Initially, this mixture was inoculated with pre‐cultured liquids of *Saccharomyces cerevisiae* SC17‐1 and *Lactiplantibacillus plantarum* LP15‐1 to facilitate facultative anaerobic fermentation over a period of 2 days. Subsequently, the mixture was inoculated with pre‐cultured liquids of *Bacillus subtilis* BS15‐3 for aerobic fermentation, also lasting 2 days. The fermentation was conducted at a temperature range of 25°C–32°C, with inoculum concentrations of SC17‐1, LP15‐1, and BS15‐3 at 1 × 10^6^, 1 × 10^6^, 1 × 10^7^ CFU/g, respectively. Upon completion of the fermentation, the FCSM samples were dried using a fluidized bed at temperatures below 55°C.

### 2.2 Chemical Analysis of CSM and FCSM

FCSM samples were dried at 55°C for 24 h, and subsequently ground to pass through a 0.425‐mm mesh screen for subsequent chemical analysis. The moisture, CP, ether extract, CF, crude ash, and free gossypol levels in both CSM and FCSM were analyzed in accordance with the National Standards of the People's Republic of China: GB/T 6435‐2014, GB/T 6432‐2018, GB/T 6433‐2006, GB/T 6434‐2022, GB/T 6438‐2007 and GB/T 6433‐2006 (Yao et al. 2024). The acid‐soluble protein of CSM and FCSM was determined by the trichloroacetic acid method (NY/T 3801‐2020). Moisture content was measured by heating the samples to a constant weight at 105°C in an electric forced‐air oven (DHG‐9140AL, Beijing Luxi Technology Co. Ltd., China), and then the dry matter content was calculated. The gross energy (GE), CP, CF, EE, and crude ash contents of CSM and FCSM were determined using an isothermal auto‐calorimeter (C6000, IKA, Germany), an automatic Kjeldahl apparatus (Kjeltec8400, Foss, Denmark), a polyester mesh bag‐automated fiber analyzer (A2000, ANKOM, USA), an automatic Soxhlet extractor (Soxtec 8000, Foss, Denmark), and a muffle furnace (TXA9, pyramid, China), respectively. To determine the content of *L*‐lactic acid, 3 g of each CSM or FCSM sample was transferred to a 50‐ml centrifuge tube with 27 mL of distilled water. The mixture was agitated on a rotary shaker at 25°C and 180 rpm for 2 h. Subsequently, the suspension was centrifuged at 4000 rpm for 20 min, and the supernatant was analyzed using a biosensor (SBA‐40E, China). The amino acid compositions of the samples were determined by an automated amino acid analyzer (A300, mwmbraPure, GER) following hydrolysis with 6 M HCl at 110°C for 24 h or with 4 M LiOH at 110°C for 20 h.

### 2.3 Experimental Design and Diets

Experiment 1: A total of 126 one‐day‐old Arbor Acres (AA) broilers were randomly assigned to three dietary treatments: CSM, FCSM, and nitrogen‐free diet (NFD), with six replicates per treatment and seven birds per replicate. During Days 1 to 24, all birds were administered a basal diet, after which they were transitioned to experimental diets starting on Day 25. Comprehensive details of the dietary compositions and nutritional profiles are presented in Table S1. At 25 days of age, the broilers were provided with either test diets or a NFD. On Day 28, following a 4‐h fasting period, the birds were allowed 1 h of ad libitum feeding, succeeded by a 3‐h withholding period prior to slaughter. The distal half of the posterior ileum was collected, and the digesta were flushed with distilled water, pooled per replicate, and stored at −20°C. The samples were then freeze‐dried, equilibrated at room temperature for 24 h, homogenized within replicates, and ground for subsequent analysis.

Experiment 2: Using a single‐factor design, 175 AA broilers were randomly assigned to 5 treatments based on body weight: (1) control group (corn‐SBM diet), (2) 6% CSM group (replacing 20% of SBM), (3) 6% FCSM group (replacing 20% of SBM), (4) 9% FCSM group (replacing 30% of SBM), and (5) 12% FCSM group (replacing 40% of SBM). The experiment was conducted using a completely randomized design, incorporating five replicates per treatment and seven birds per replicate. Detailed formulations of the diets and their nutritional compositions are presented in Table 1. The basal diet was formulated to align with the nutrient requirements as recommended by the National Research Council (NRC 1994).

**TABLE 1:** Ingredient composition and nutrient contents of the experimental diets (%, air‐dry basis).

Items	Starter (Days 1 to 21)					Finisher (Days 22 to 42)				
	CON	6% CSM	6% FCSM	9% FCSM	12% FCSM	CON	6% CSM	6% FCSM	9% FCSM	12% FCSM
Corn	56.51	56.61	57.90	58.61	59.30	56.85	57.00	58.28	58.98	59.68
Soybean meal	30.00	24.00	24.00	21.00	18.00	30.00	24.00	24.00	21.00	18.00
CSM	0.00	6.00	0.00	0.00	0.00	0.00	6.00	0.00	0.00	0.00
FCSM	0.00	0.00	6.00	9.00	12.00	0.00	0.00	6.00	9.00	12.00
Corn gluten meal	6.56	6.40	5.71	5.30	4.87	5.44	5.28	4.60	4.19	3.77
Soybean oil	2.62	2.56	2.00	1.68	1.37	4.21	4.12	3.57	3.25	2.94
Dicalcium phosphate	1.78	1.80	1.80	1.80	1.78	1.48	1.50	1.50	1.49	1.50
Limestone	1.33	1.34	1.32	1.32	1.35	1.26	1.26	1.24	1.25	1.24
Sodium chloride	0.30	0.30	0.30	0.30	0.30	0.30	0.30	0.30	0.30	0.30
Premix^a^	0.23	0.23	0.23	0.23	0.23	0.23	0.23	0.23	0.23	0.23
Choline chloride	0.10	0.10	0.10	0.10	0.10	0.10	0.10	0.10	0.10	0.10
*DL*‐methionine	0.23	0.23	0.23	0.22	0.22	0.13	0.13	0.12	0.12	0.12
*L*‐Lysine	0.26	0.33	0.32	0.34	0.37	0.00	0.07	0.06	0.08	0.11
Threonine	0.08	0.10	0.09	0.10	0.11	0.00	0.01	0.00	0.01	0.01
Total, %	100	100	100	100	100	100	100	100	100	100
Nutrient content,^b^ %										
ME (kcal/kg)	2997	2997	2997	2997	2997	3097	3097	3097	3097	3097
Crude protein	22.00	22.00	22.00	22.00	22.00	21.00	21.00	21.00	21.00	21.00
Calcium	1.00	1.00	1.00	1.00	1.00	0.90	0.90	0.90	0.90	0.90
Total phosphorus	0.62	0.65	0.66	0.68	0.69	0.56	0.59	0.60	0.61	0.63
Lysine	1.20	1.20	1.20	1.20	1.20	1.00	1.00	1.00	1.00	1.00
Methionine	0.50	0.50	0.50	0.50	0.50	0.40	0.40	0.40	0.40	0.40
FG (mg/kg)	—	98.26	24.99	37.48	49.98	—	98.26	24.99	37.48	49.98

Abbreviations: CON, corn‐soybean basal diet; CSM, cottonseed meal; FCSM, fermented cottonseed meal; FG, free gossypol.

^a^ The premix provided the following per kg of diets: vitamin A, 9500 IU; vitamin D_3_, 62.5 ug; vitamin K_3_, 2.65 mg; vitamin B_12_, 0.025 mg; vitamin B_2_, 6 mg; vitamin E, 30 IU; biotin, 0.0325 mg; folic acid, 1.25 mg; pantothenic acid 12 mg; nicotinic acid, 50 mg; Cu, 8 mg; Zn, 75 mg; Fe, 80 mg; Mn, 100 mg; Se, 0.15 mg; I, 0.35 mg.

^b^ Nutrient content were calculated values.

The trial employed a four‐tier cage system with the following management conditions: the brooding temperature was maintained at 32°C–35°C during the first week and was gradually reduced to 24°C thereafter, with a lighting schedule of 23 h per day. Birds were vaccinated following standard protocols and were given ad libitum access to feed and water. Feeding was scheduled at 07:00, 13:00, and 17:00 daily, with regular health monitoring and mortality recording, as described by Song et al. (2016). On Days 21 and 42 of the trial, one bird per replicate was randomly selected for blood collection from the wing vein into coagulation tubes. Serum was obtained by centrifuging at 2000 × g (4°C, 15 min) and stored at −20°C for subsequent analysis. Following blood collection, birds were euthanized by CO_2_ asphyxiation and exsanguinated. The ileal samples were preserved in formalin for histological analysis. Cecal digesta was divided into two aliquots: one was snap‐frozen in liquid nitrogen and stored at −80°C for subsequent SCFA assays, and the other was maintained at 4°C for microbial analysis.

### 2.4 Ileal Amino Acid Digestibility

The apparent and standardized digestibility coefficients for amino acids in CSM and FCSM within the ileum of broilers were determined using the proportions of a titanium marker in the diet. A NFD was formulated to evaluate the basal endogenous loss of amino acids, with titanium dioxide (TiO_2_) employed as the digestibility marker. The calculation for ileal digestibility was as follows:

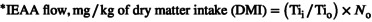

$$\begin{array}{c} \#\text{The apparent ileal digestibility of amino acid}\;\left(\mathrm{AID},\%\right)\\ \;=\left[1-\left({\mathrm{Ti}}_{i}/{\mathrm{Ti}}_{o}\right)\times N_{o}/N_{i}\right]\times 100 \end{array}$$

$$\begin{array}{c} \#\text{Standardized ileal digestibility of amino acid}\;\left(\mathrm{SID},\%\right)=\left(\mathrm{AID},\%\right)\\ \;+\left[100\times \left(\text{Basal IEAA flow}/\text{Amino acids in diet}\right)\right] \end{array}$$

Ti_i_ indicates the amount of titanium present in the diet, and Ti_o_ denotes the titanium concentration in the ileal digesta. *N*_i_ refers to the measured amino acid levels in the diet, and *N*_o_ signifies the amino acid concentration in the ileal digesta (Teng et al. 2021). *Assessed by the NFD diet, #Assessed by the regular diet.

### 2.5 Growth Performance

On Days 21 and 42 of the trial, after a 12‐h fasting period, all birds were individually weighed and feed intake was recorded on a per‐replicate basis. Performance parameters including average daily gain (ADG), average daily feed intake (ADFI), and feed conversion ratio (FCR) were calculated for three growth phases: Days 1 to 21, Days 22 to 42, and Days 1 to 42.

### 2.6 Immune Performance

The serum concentrations of immunoglobulin A (IgA), immunoglobulin G (IgG), and immunoglobulin M (IgM) were determined using chicken‐specific ELISA kits (Beijing Huaying Biotechnology Co. Ltd.; catalog numbers: HY‐N0048 for IgA, HY‐N0050 for IgG, HY‐N0049 for IgM) following the manufacturer's protocols (Song et al. 2022).

### 2.7 Intestinal Morphometric Measurement

To assess the morphology, a segment approximately 3 cm in length was excised from the central region of the ileum. The intestinal samples were subsequently rinsed with physiological saline and fixed in 10% buffered formalin for a period of 24 h. The samples underwent sequential dehydration through a graded ethanol series (50%, 70%, 80%, 96%, and 100%), followed by clearing in xylene and embedding in paraffin. Sections with a thickness of 5 μm were prepared using a microtome, with three cross‐sections obtained per sample. These sections were mounted on glass slides and stained with hematoxylin and eosin. Villus height (VH) and crypt depth (CD) were quantified using an image analyzer (BOND‐III, Leica Microsystems, Cambridge, UK), with five replicates analyzed per treatment to evaluate intestinal morphology (Kasaeizadeh et al. 2025).

### 2.8 Cecal Microbiota

Approximately 1 g of digesta was homogenized in 9 mL of sterile PBS, achieving a 1:10 dilution, in a sterile 15‐mL centrifuge tube, followed by serial 10‐fold dilutions. Aliquots were then plated on selective media for bacterial enumeration: (1) coliforms on eosin methylene blue agar under aerobic conditions at 37°C for 24 h; (2) *Salmonella* on Salmonella Shigella agar under aerobic conditions at 37°C for 24 h; and (3) lactic acid bacteria on de Man, Rogosa, and Sharpe agar under anaerobic conditions at 37°C for 48 h. The data for microbiota were shown as log_10_ CFU per gram of caecal digesta (Song et al. 2022).

### 2.9 Cecal SCFA Analysis

A 0.5 g sample was homogenized with ultrapure water at a ratio of 1:5 (w:v) using a vortex mixer for 30 min, followed by centrifugation to isolate the supernatant. This extraction procedure was repeated one to two times, and the combined supernatants were diluted to a final volume of 10 mL in a colorimetric tube. A portion of the extract underwent centrifugation at 15,000 rpm for 15 min, and the resulting supernatant was treated with 25% metaphosphoric acid in a 9:1 (v:v) ratio for fixation over a period of 3 h.

The analysis of SCFA was carried out by gas chromatography using an Agilent 6890 N system (Agilent Technologies, MA, USA) equipped with a hydrogen flame‐ionization detector (FID) and an Agilent J&W DB‐FFAP capillary column (30 m × 0.32 mm × 0.25 μm). The injection temperature was 200°C. Samples were injected at a 10:1 split ratio mode in 1 μL aliquots. Helium was used as the carrier gas at flow rate of 0.8 mL/min. The oven temperature program and FID condition was set with reference to Fruci et al. (2023).

### 2.10 Statistical Analysis

Data processing was conducted using Microsoft Excel (Microsoft Excel 2010 v14.0) to compute group means, standard deviations, and coefficients of variation. Statistical significance was assessed via one‐way ANOVA followed by Duncan's multiple range test, utilizing SPSS software (version 23.0; IBM Corp.). Results are expressed as mean ± SE, with a significance threshold set at *p* < 0.05.

## 3 Results

### 3.1 Chemical Composition of CSM and FCSM

As illustrated in Table 2, the results indicated that CSM fermented with *Bacillus subtilis* BS15‐3, *Saccharomyces cerevisiae* SC17‐1, and *Lactiplantibacillus plantarum* LP15‐1 exhibited significantly higher levels of CP (44.66% vs. 49.29%), acid‐soluble protein (2.41% vs. 5.95%), *L*‐lactic acid (0.01 vs. 1.50 g/L), and GE (3819 vs. 4500 kcal/kg) compared to CSM (*p* < 0.05). Furthermore, the free gossypol content in FCSM was significantly reduced by 74.57% compared to CSM, decreasing from 1637.61 mg/kg to 416.5 mg/kg. Fermentation of CSM also resulted in a reduction of CF content (15.72% compared to 13.6%) (*p* < 0.05). However, no significant differences were observed in other chemical compositions. (*p* > 0.05).

**TABLE 2:** Chemical composition of cottonseed meal (CSM) and fermented cottonseed meal (FCSM) (%, air‐dry basis).

Item	CSM	FCSM	*p*
Dry matter	90.54 ± 0.37	92.01 ± 0.74	0.174
Crude protein	44.66 ± 0.29^a^	49.29 ± 0.20^b^	< 0.001
Crude fiber	15.72 ± 0.44^b^	13.60 ± 0.31^a^	0.009
Ether extract	0.63 ± 0.01	0.56 ± 0.03	0.079
Ash	7.33 ± 0.05	7.32 ± 0.01	0.930
Acid soluble protein	2.41 ± 0.04^b^	5.95 ± 0.14^a^	< 0.001
Free gossypol (mg/kg)	1637.61 ± 43.06^a^	416.5 ± 13.40^b^	< 0.001
*L‐*lactic acid (g/L)	0.01 ± 0.00^b^	1.50 ± 0.09^a^	< 0.001
Gross energy (kcal/kg)	3819 ± 70^b^	4500 ± 40^a^	< 0.001
Aspartate	3.00 ± 0.51	3.00 ± 0.55	0.995
Threonine	1.22 ± 0.16	1.34 ± 0.10	0.555
Serine	1.48 ± 0.26	1.73 ± 0.03	0.431
Glutamate	7.72 ± 0.44	8.00 ± 0.51	0.697
Proline	1.61 ± 0.08	1.71 ± 0.08	0.452
Glycine	1.66 ± 0.04	1.69 ± 0.06	0.700
Alanine	1.58 ± 0.06	1.63 ± 0.09	0.679
Cystine	0.25 ± 0.19	0.32 ± 0.18	0.795
Valine	1.64 ± 0.14	2.04 ± 0.03	0.094
Methionine	0.36 ± 0.11	0.65 ± 0.25	0.349
Isoleucine	1.26 ± 0.03	1.27 ± 0.07	0.930
Leucine	2.42 ± 0.05	2.61 ± 0.15	0.275
Tyrosine	1.00 ± 0.10	1.47 ± 0.43	0.345
Phenylalanine	2.11 ± 0.02	1.81 ± 0.32	0.442
Histidine	1.20 ± 0.08	1.33 ± 0.13	0.444
Lysine	1.75 ± 0.08	1.75 ± 0.12	0.995
Arginine	4.64 ± 0.31	4.79 ± 0.29	0.741
Tryptophan	0.54 ± 0.00	0.53 ± 0.01	0.995
Total free amino acids	35.43 ± 2.48	37.64 ± 2.18	0.539

*Note:* Within each row, values with different superscripts were different, *p* < 0.05.

Abbreviations: CSM, cottonseed meal; FCSM, fermented cottonseed meal.

### 3.2 Amino Acid Ileal Digestibility Analysis of CSM and FCSM

Table 3 provides a summary of the effects of fermentation on amino acid digestibility in CSM. The FCSM group exhibited a significant increase in the AID of valine (17.82% vs. 58.32%) and an enhancement in the SID of glutamate (80.58% vs. 85.29%) and valine (52.06% vs. 84.25%) (*p* < 0.05). No significant alterations were observed in either AID or SID values for the other amino acids (*p* > 0.05).

**TABLE 3:** Apparent ileal digestibility (AID) and standardized ileal digestibility (SID) of amino acids of CSM and FCSM in broilers (%, DM basis).

Item	AID			SID		
	CSM	FCSM	*p*	CSM	FCSM	*p*
Aspartate	72.33 ± 1.15	71.36 ± 0.35	0.491	75.87 ± 1.16	76.38 ± 0.94	0.750
Threonine	60.36 ± 2.58	60.95 ± 0.74	0.846	69.50 ± 3.04	73.21 ± 1.99	0.374
Serine	74.02 ± 0.64	72.79 ± 0.77	0.291	79.14 ± 0.49	79.81 ± 1.60	0.732
Glutamate	77.66 ± 1.57	81.93 ± 0.26	0.109	80.58 ± 1.42^b^	85.29 ± 0.43^a^	0.034
Proline	70.75 ± 0.40	70.21 ± 0.80	0.589	76.36 ± 0.55	77.26 ± 1.58	0.638
Glycine	69.03 ± 0.85	68.76 ± 1.51	0.885	73.54 ± 0.49	74.87 ± 1.38	0.443
Alanine	69.52 ± 0.72	68.57 ± 0.93	0.470	74.25 ± 0.72	74.92 ± 1.38	0.695
Cystine	71.88 ± 1.78	72.03 ± 1.47	0.951	77.57 ± 2.22	80.48 ± 1.14	0.327
Valine	17.82 ± 2.13^b^	58.32 ± 3.10^a^	0.001	52.06 ± 2.04^b^	84.25 ± 3.26^a^	0.002
Methionine	70.93 ± 5.95	71.13 ± 5.58	0.981	75.29 ± 5.90	76.39 ± 5.58	0.899
Isoleucine	68.37 ± 1.28	64.80 ± 1.26	0.117	73.70 ± 0.86	71.89 ± 0.98	0.239
Leucine	71.40 ± 0.71	76.46 ± 2.39	0.160	74.32 ± 0.68	80.35 ± 2.29	0.109
Tyrosine	72.55 ± 1.39	73.84 ± 0.51	0.457	73.22 ± 1.35	74.73 ± 0.40	0.383
Phenylalanine	82.03 ± 0.49	82.42 ± 0.38	0.572	83.50 ± 0.49	84.38 ± 0.27	0.214
Histidine	73.31 ± 0.96	75.89 ± 1.77	0.289	77.36 ± 1.07	81.10 ± 1.88	0.178
Lysine	63.82 ± 4.08	57.71 ± 0.94	0.272	68.55 ± 3.95	64.23 ± 0.91	0.389
Arginine	87.60 ± 0.64	87.60 ± 0.79	0.997	89.03 ± 0.56	89.57 ± 0.71	0.586

*Note:* Within each row, values with different superscripts were different, *p* < 0.05.

Abbreviations: AID, apparent ileal digestibility; CSM, cottonseed meal; FCSM, fermented cottonseed meal; SID, standardized ileal digestibility.

### 3.3 Effects of Different Levels of FCSM on Growth Performance

The impact of varying levels of FCSM on growth performance is detailed in Table 4. At 21 days of age, the group receiving 9% FCSM demonstrated a significantly higher BW compared to the group receiving 6% CSM (*p* < 0.05). However, no statistically significant differences in BW were observed between the groups at 42 days of age. From Days 1 to 21, the group receiving 6% FCSM exhibited higher ADFI, and the group receiving 9% FCSM showed higher ADG compared to the 6% CSM group (*p* < 0.05). During the period from Days 22 to 42, the FCR of the 12% FCSM group increased significantly compared to the control group (*p* < 0.05). No significant differences in ADG and ADFI were observed among all groups during the finisher phase (Days 22 to 42) and the entire experimental period (Days 1 to 42) (*p* > 0.05).

**TABLE 4:** Effects of different levels of FCSM on growth performance of broilers.

Items	CON	6% CSM	6% FCSM	9% FCSM	12% FCSM	*p*
Day 21 BW (g)	600.12 ± 6.93^ab^	596.93 ± 17.42^b^	622.09 ± 9.73^ab^	642.16 ± 17.78^a^	632.35 ± 12.58^ab^	0.019
Day 42 BW (g)	2112.35 ± 32.42	2022.11 ± 39.23	2023.54 ± 39.60	2138.30 ± 64.95	2070.36 ± 59.92	0.304
Day 1 to 21						
ADG (g/day)	26.69 ± 0.33^ab^	26.52 ± 0.83^b^	27.72 ± 0.46^ab^	28.68 ± 0.85^a^	28.22 ± 0.59^ab^	0.019
ADFI (g/day)	36.20 ± 0.58^b^	35.81 ± 1.83^b^	39.70 ± 0.89^a^	38.49 ± 0.48^ab^	38.35 ± 0.86^ab^	0.047
FCR (g/g)	1.36 ± 0.03	1.41 ± 0.04	1.43 ± 0.03	1.35 ± 0.04	1.36 ± 0.04	0.382
Day 22 to 42						
ADG (g/day)	72.01 ± 1.26	67.87 ± 1.52	66.73 ± 1.90	71.48 ± 2.59	68.48 ± 2.54	0.304
ADFI (g/day)	109.14 ± 1.55	110.26 ± 3.31	108.10 ± 1.36	111.19 ± 2.67	112.11 ± 2.72	0.400
FCR (g/g)	1.52 ± 0.01^b^	1.62 ± 0.05^ab^	1.63 ± 0.04^ab^	1.56 ± 0.03^b^	1.64 ± 0.03^a^	0.038
Day 1 to 42						
ADG (g/day)	49.35 ± 0.77	47.20 ± 0.93	47.23 ± 0.94	49.96 ± 1.55	48.35 ± 1.42	0.368
ADFI (g/d)	72.67 ± 0.92	73.04 ± 0.90	73.90 ± 0.49	74.84 ± 0.48	74.90 ± 1.61	0.686
FCR (g/g)	1.47 ± 0.01	1.55 ± 0.04	1.57 ± 0.03	1.50 ± 0.04	1.52 ± 0.05	0.417

*Note:* Data were means from five replicates per treatment, each treatment included 35 birds. Within each row, values with different superscripts were different, *p* < 0.05.

Abbreviations: ADFI, average daily feed intake; ADG, average daily gain; BW, body weight; FCR, feed conversion ratio; CON, corn‐soybean basal diet; CSM, cottonseed meal; FCSM, fermented cottonseed meal.

### 3.4 Effects of Different Levels of FCSM on Immune Performance

The effects of different inclusion levels of FCSM on immune performance of broilers were presented in Table 5. Dietary supplementation with 6% and 9% FCSM significantly increased serum concentrations of IgA, IgM, and IgG in broilers at both 21 and 42 days of age compared to the control group (*p* < 0.05). Furthermore, dietary supplementation with 9% FCSM significantly increased serum concentrations of IgA, IgM, and IgG in broilers at 21 days of age compared to the group receiving 6% CSM (*p* < 0.05).

**TABLE 5:** Effects of different levels of FCSM on serum immunoglobulins of broilers.

Items	CON	6% CSM	6% FCSM	9% FCSM	12% FCSM	*p*
Day 21						
IgA (g/L)	1.65 ± 0.05^d^	2.18 ± 0.25^bc^	2.62 ± 0.17^ab^	2.84 ± 0.20^a^	2.08 ± 0.07^cd^	< 0.001
IgG (g/L)	3.00 ± 0.03^c^	4.45 ± 0.16^b^	4.74 ± 0.28^ab^	5.52 ± 0.46^a^	4.27 ± 0.22^b^	< 0.001
IgM (g/L)	1.33 ± 0.10^c^	1.66 ± 0.05^bc^	1.73 ± 0.11^b^	2.26 ± 0.22^a^	1.59 ± 0.04^bc^	< 0.001
Day 42						
IgA (g/L)	1.41 ± 0.13^c^	2.09 ± 0.04^b^	2.49 ± 0.26^a^	2.18 ± 0.03^ab^	2.13 ± 0.03^ab^	< 0.001
IgG (g/L)	2.61 ± 0.33^b^	4.23 ± 0.06^a^	4.48 ± 0.07^a^	4.37 ± 0.18^a^	4.16 ± 0.20^a^	< 0.001
IgM (g/L)	1.30 ± 0.08^b^	1.82 ± 0.03^a^	2.14 ± 0.31^a^	1.88 ± 0.05^a^	1.80 ± 0.04^a^	0.022

*Note:* Data were means from five replicates per treatment. Within each row, values with different superscripts were different, *p* < 0.05.

Abbreviations: CON, corn‐soybean basal diet; CSM, cottonseed meal; FCSM, fermented cottonseed meal; IgA, immunoglobulin A; IgG, immunoglobulin G; IgM, immunoglobulin M.

### 3.5 Effects of Different Levels of FCSM on Ileal Morphology

The findings pertaining to ileal morphology in broilers are detailed in Table 6. At 21 days of age, dietary supplementation with 9% and 12% FCSM group resulted in a statistically significant increase in ileal VH compared to the control and 6% CSM groups (*p* < 0.05). The VH‐to‐CD ratio (VH/CD ratio) was notably higher in the 6% FCSM group than in the 6% CSM group, while the 12% FCSM group demonstrated deeper crypts than both the control and 6% CSM groups (*p* < 0.05). By 42 days, the 6% FCSM group exhibited significant morphological enhancements, characterized by reduced CD and an elevated VH/CD ratio relative to the control and 6% CSM group (*p* < 0.05).

**TABLE 6:** Effects of different levels of FCSM on intestinal morphology of broilers.

Items	CON	6% CSM	6% FCSM	9% FCSM	12% FCSM	*p*
Day 21						
Villus height (μm)	422.34 ± 25.19^b^	341.71 ± 21.28^c^	404.72 ± 16.20^bc^	549.04 ± 33.84^a^	512.20 ± 28.48^a^	0.004
Crypt depth (μm)	68.49 ± 8.34^b^	72.13 ± 2.65^b^	65.28 ± 3.88^b^	79.50 ± 6.53^ab^	97.82 ± 4.68^a^	0.005
VH/CD ratio	5.86 ± 0.53^ab^	5.17 ± 0.50^b^	7.11 ± 0.55^a^	6.07 ± 0.36^ab^	5.32 ± 0.19^b^	0.034
Day 42						
Villus height (μm)	901.95 ± 85.28	778.07 ± 52.25	883.81 ± 47.98	805.25 ± 19.42	867.70 ± 69.21	0.506
Crypt depth (μm)	149.15 ± 2.54^a^	141.61 ± 5.48^ab^	108.15 ± 5.14^c^	128.71 ± 4.24^b^	139.24 ± 4.02^ab^	< 0.001
VH/CD ratio	6.92 ± 0.88^b^	5.58 ± 0.53^c^	8.15 ± 0.54^a^	6.46 ± 0.24^b^	5.55 ± 0.33^c^	0.038

*Note:* Data were means from five replicates per treatment. Within each row, values with different superscripts were different, *p* < 0.05.

Abbreviations: CON, corn‐soybean basal diet; CSM, cottonseed meal; FCSM, fermented cottonseed meal; VH/CD ratio, villus height‐to‐crypt depth ratio.

### 3.6 Effects of Different Levels of FCSM on Cecal Microbiota

Table 7 presents the impact of varying levels of FCSM on the microbial population in the cecum of broiler. At 21 days of age, the 9% FCSM group showed a significant reduction in coliform counts compared to the control group (*p* < 0.05). By 42 days of age, the 9% FCSM group further demonstrated a significant decrease in coliform counts relative to both the control and 6% CSM groups (*p* < 0.05). Importantly, at both 21 and 42 days of age, the 9% FCSM group exhibited significant probiotic effects, as evidenced by a marked increase in lactic acid bacteria populations and concurrently reduced *Salmonella* counts compared to the control and 6% CSM groups (*p* < 0.05).

**TABLE 7:** Effects of different levels of FCSM on the number of microorganisms in the intestinal contents of broilers (log_10_ CFU/g).

Items	CON	6% CSM	6% FCSM	9% FCSM	12% FCSM	*p*
Day 21						
*Lactic acid bacteria*	9.97 ± 0.07^b^	10.39 ± 0.17^b^	11.09 ± 0.14^a^	11.22 ± 0.06^a^	10.47 ± 0.19^b^	< 0.001
*Coliforms*	7.06 ± 0.22^a^	6.57 ± 0.21^ab^	6.61 ± 0.06^ab^	5.98 ± 0.29^b^	6.50 ± 0.32^ab^	0.044
*Salmonella*	6.15 ± 0.15^a^	5.86 ± 0.24^ab^	5.45 ± 0.25^abc^	4.71 ± 0.22^c^	5.27 ± 0.27^bc^	0.006
Day 42						
*Lactic acid bacteria*	9.89 ± 0.08^c^	9.69 ± 0.11^c^	10.41 ± 0.29^b^	11.11 ± 0.15^a^	10.85 ± 0.14^ab^	< 0.001
*Coliforms*	7.80 ± 0.07^a^	7.88 ± 0.09^a^	7.21 ± 0.06^b^	6.67 ± 0.23^bc^	6.34 ± 0.15^c^	< 0.001
*Salmonella*	6.94 ± 0.06^b^	7.55 ± 0.09^a^	5.93 ± 0.19^c^	5.93 ± 0.08^c^	5.18 ± 0.23^d^	< 0.001

*Note:* Data were means from five replicates per treatment. Within each row, values with different superscripts were different, *p* < 0.05.

Abbreviations: CON, corn‐soybean basal diet; CSM, cottonseed meal; FCSM, fermented cottonseed meal.

### 3.7 Effects of Different Levels of FCSM on Cecal SCFA

The effects of different levels of FCSM on cecal SCFA of broiler were indicated in Table 8. At 21 days of age, the 6% FCSM group exhibited significantly higher concentrations of acetate, propionate, and total SCFA in the cecal contents compared to both the control and 6% CSM groups (*p* < 0.05). Additionally, the 9% FCSM group demonstrated a significant increase in valerate content (*p* < 0.05). Butyrate levels were significantly elevated in the 6% FCSM group relative to the control, whereas isovalerate concentrations were significantly lower in both the 9% and 12% FCSM groups compared to the control (*p* < 0.05). At 42 days of age, the 9% FCSM group showed significantly reduced butyrate levels compared to the control (*p* < 0.05). Furthermore, relative to the 6% CSM group, the 6% FCSM group had significantly elevated propionate, isovalerate, and total volatile fatty acid levels, while the 9% FCSM group exhibited a marked increase in propionate, isobutyrate, isovalerate levels at Day 42 (*p* < 0.05).

**TABLE 8:** Effects of different levels of FCSM on short‐chain fatty acids in the intestinal contents of broilers (mg/g).

Items	CON	6% CSM	6% FCSM	9% FCSM	12% FCSM	*p*
Day 21						
Acetate	4.26 ± 0.57^b^	4.68 ± 0.16^b^	5.92 ± 0.38^a^	4.47 ± 0.03^b^	5.80 ± 0.12^a^	0.011
Propionate	0.59 ± 0.03^c^	0.66 ± 0.03^bc^	0.83 ± 0.02^a^	0.73 ± 0.02^b^	0.72 ± 0.04^b^	< 0.001
Isobutyrate	0.08 ± 0.01^ab^	0.09 ± 0.01^ab^	0.10 ± 0.01^a^	0.09 ± 0.01^ab^	0.07 ± 0.01^b^	0.024
Butyrate	0.74 ± 0.07^b^	1.27 ± 0.12^a^	1.64 ± 0.15^a^	0.96 ± 0.09^ab^	1.04 ± 0.01^ab^	< 0.001
Isovalerate	0.15 ± 0.01^a^	0.12 ± 0.01^bc^	0.13 ± 0.01^ab^	0.11 ± 0.01^cd^	0.09 ± 0.01^d^	0.002
Valerate	0.14 ± 0.02^b^	0.11 ± 0.01^b^	0.12 ± 0.01^b^	0.19 ± 0.01^a^	0.14 ± 0.01^b^	0.019
Total SCFA	5.96 ± 0.49^c^	6.93 ± 0.22^c^	8.74 ± 0.34^a^	6.55 ± 0.22^c^	7.86 ± 0.14^ab^	< 0.001
Day 42						
Acetate	5.17 ± 0.19	4.25 ± 0.30	5.54 ± 0.66	4.95 ± 0.91	5.30 ± 0.29	0.476
Propionate	1.27 ± 0.09^a^	0.66 ± 0.03^c^	1.10 ± 0.05^ab^	1.01 ± 0.18^a^	0.79 ± 0.09^bc^	0.003
Isobutyrate	0.13 ± 0.01^ab^	0.11 ± 0.01^b^	0.15 ± 0.01^ab^	0.19 ± 0.03^a^	0.11 ± 0.01^b^	0.006
Butyrate	1.50 ± 0.23^a^	0.95 ± 0.09^ab^	1.18 ± 0.23^ab^	0.83 ± 0.10^b^	1.35 ± 0.13^ab^	0.029
Isovalerate	0.16 ± 0.01^ab^	0.13 ± 0.01^bc^	0.19 ± 0.01^a^	0.17 ± 0.01^a^	0.12 ± 0.01^c^	0.005
Valerate	0.19 ± 0.03	0.18 ± 0.03	0.19 ± 0.02	0.19 ± 0.01	0.18 ± 0.03	0.994
Total SCFA	8.44 ± 0.28^a^	6.28 ± 0.42^b^	8.35 ± 0.8^a^	7.35 ± 0.43^ab^	7.86 ± 0.43^a^	0.022

*Note:* Data were means from five replicates per treatment. Within each row, values with different superscripts were different, *p* < 0.05.

Abbreviations: CON, corn‐soybean basal diet; CSM, cottonseed meal; FCSM, fermented cottonseed meal; SCFA, short‐chain fatty acids.

## 4 Discussion

China has substantial resources of CSM, which has long been considered a viable alternative to SBM (Yan et al. 2025). Nonetheless, the presence of the anti‐nutritional factors, such as free gossypol, negatively impacts animal health and reproductive performance, thereby restricting its use in animal feed and leading to suboptimal utilization of protein resources (Ashayerizadeh, Jazi, Rezvani, et al. 2024; Xie et al. 2024). Numerous studies have demonstrated that microbial fermentation can effectively degrade free gossypol and enhance the nutritional quality of CSM, although the effectiveness varies considerably depending on the microbial strains and fermentation processes utilized (Han et al. 2022; Ashayerizadeh, Jazi, Sharifi, et al. 2024; Liu et al. 2024). In this study, a mixed culture of *Bacillus subtilis* BS15‐3, *Saccharomyces cerevisiae* SC17‐1, and *Lactiplantibacillus plantarum* LP15‐1 was employed to ferment CSM. The removal rate of free gossypol exceeded 74.6%, which was accompanied by a significant increase in CP, acid‐soluble protein, and GE, as well as a reduction in CF content. These findings were aligned with those reported by Wang et al. (2017), who similarly documented a reduction in gossypol and CF, alongside an increase in acid‐soluble protein after fermenting CSM with *Bacillus subtilis* ST‐141 and *yeast* N5. The observed enhancement in the GE content of FCSM is likely attributable to the elevated CP levels, given that protein inherently possesses a higher energy value compared to non‐structural carbohydrates (Wu et al. 2020). The augmentation in CP content can be attributed to a concentration effect occurring during fermentation (Wu et al. 2020). The increase in acid‐soluble protein is plausibly a consequence of the breakdown of macromolecular proteins into smaller peptides and free amino acids, facilitated by proteases secreted by microorganisms (Li et al. 2022). The observed reduction in CF may be linked to the production of cellulases as a result of microbial activity. Furthermore, the degradation of free gossypol may be facilitated either through direct decomposition by microbial enzymes or through binding with proteins or amino acids, resulting in the formation of bound gossypol (Xie et al. 2024). This study further demonstrated a significant enhancement in the SID of glutamate and valine following fermentation, which may be linked to lower CF levels, as fiber reduction generally promotes amino acid digestibility (Zhuo et al. 2023). Additionally, the optimization of protein structure and the elimination of anti‐nutritional factors facilitated the release and absorption of amino acids. The increased production of *L*‐lactic acid during fermentation was mainly due to the growth of *Lactiplantibacillus plantarum* LP15‐1, which produced *L*‐lactic acid by utilizing glucose in CSM, in addition, the increased *L*‐lactic acid content in FCSM may be one of the factors contributing to the improved amino acid digestibility (Wu et al. 2020).

Based on the demonstrated nutritional improvements of FCSM, this study further systematically evaluated its potential as a SBM substitute in broiler diets, with a focus on production performance, immune performance, intestinal morphology, microbial composition, and SCFA metabolism. The results of this study revealed that the 21‐day body weight and ADG in the group receiving 9% FCSM were significantly greater than those observed in the group receiving 6% CSM. This observation was consistent with the work of Ashayerizadeh, Jazi, Sharifi, et al. (2024), who demonstrated that FCSM significantly enhances body weight, daily gain, and FCR in broilers, likely attributable to reduced free gossypol content. Additionally, the present study indicated that FCSM offered superior nutritional value compared to CSM and positively influences immune function, intestinal morphology, and cecal microbiota, which may further contribute to improved growth performance (Niu et al. 2021). Conversely, Wang et al. (2017) reported that dietary supplementation with FCSM did not significantly impact ADG in broilers. The divergence in findings may be attributed to differences in antinutrient factors, nutritional quality, the inclusion levels of the FCSM, and fermentation strains utilized (Xie et al. 2024). The growth‐promoting effects of FCSM may be mediated through modulation of host immunity and enhancement of intestinal health (Gong et al. 2025). To investigate this mechanism, the present study further examined changes in immune performance and intestinal health status of broilers.

Serum immunoglobulin concentration is a critical marker of humoral immune function in animals (Esen 2024). These immunoglobulins are synthesized and secreted by B cells upon activation and differentiation within lymph nodes in response to antigens or allergens, thereby playing an essential role in protecting the host against pathogenic viruses and microorganisms (Zhu et al. 2020). Previous research has indicated that the dietary inclusion of 7.5% CSM fermented with *Bacillus subtilis* ST‐141 and *yeast* N5 resulted in increased levels of IgG and IgM (Wang et al. 2017). Similarly, supplementation with 4% to 12% CSM fermented with *B. subtilis* BJ‐1 significantly elevated serum concentrations of IgA, IgG, and IgM (Esen 2024). In alignment with these findings, the current study demonstrated that incorporating 6% and 9% FCSM into the diet significantly enhanced serum levels of IgA, IgM, and IgG in broilers at both 21 and 42 days of age. This observed increase in serum immunoglobulins may be attributed to the production of short‐chain peptides and accumulation of paraprobiotics during the fermentation process, because the fermentative strains were inactivated by drying at the end of fermentation (Feng et al. 2007).

Intestinal health is intricately linked to intestinal morphology, microbial composition, and microbial metabolites, including SCFA. Key morphological indicators of intestinal health encompass VH and CD. VH is positively associated with nutrient absorption capacity; a decrease in VH can impair nutrient digestion and absorption (Ghiasvand et al. 2021; Sakwiwatkul et al. 2025). CD serves as an indicator of intestinal epithelial renewal rate: shallow crypts are typically correlated with enhanced cell maturation and secretory function, promoting villus renewal and regeneration, whereas deeper crypts may suggest mucosal atrophy and diminished absorptive capacity, potentially resulting in intestinal dysbiosis and proliferation of pathogenic bacteria such as *Escherichia coli* (Ghiasvand et al. 2021; Moreno‐Mendoza et al. 2021). The findings of this study demonstrated that, in comparison to the 6% CSM group, the 6% FCSM group exhibited significantly higher VH/CD ratio at both 21 and 42 days of age, along with a significant reduction in CD at 42 days. Additionally, the 9% FCSM group showed a significant increase in VH at 21 days. In alignment with the findings of Ashayerizadeh, Jazi, Rezvani, et al. (2024), dietary supplementation with FCSM has been shown to significantly enhance jejunal VH and the VH/CD ratio in laying hens. An elevated VH/CD ratio typically signifies an increased surface area for nutrient absorption, thereby indicating improved digestive and absorptive efficiency (Farahat et al. 2021). The augmentation of beneficial intestinal microbiota is likely a crucial factor contributing to these morphological enhancements, as optimizing microbial composition aids in suppressing pathogenic bacteria such as *Escherichia coli* , thus safeguarding intestinal structure (Soumeh et al. 2019). Wang et al. (2017) reported that FCSM supplementation markedly increased the abundance of *Lactobacilli* in the cecum of broilers when compared to diets containing CSM. This study further demonstrated that the group receiving 6% and 9% FCSM exhibited significantly higher levels of lactic acid bacteria compared to both the CSM and control groups. *Lactobacillus* has been shown to enhance intestinal barrier integrity, modulate immune responses, and reduce intestinal inflammation, providing a plausible mechanism for the FCSM‐induced improvements in intestinal morphology (Li et al. 2024; Song et al. 2025).

The proliferation of *Lactobacillus* facilitates the production of lactic acid and SCFA, subsequently lowering the pH of the digestive tract and establishing an optimal environment for digestive processes (Ashayerizadeh, Jazi, Sharifi, et al. 2024). This represents one of the reasons for which FCSM enhances production performance. SCFA not only function as energy substrates but also play a role in modulating immune responses and maintaining metabolic balance by activating G protein‐coupled receptors (such as FFAR2/FFAR3 and GPR109A) or inhibiting histone deacetylases (Ali et al. 2022). Additionally, SCFA contribute significantly to the improvement of intestinal morphology by enhancing barrier function through the promotion of tight junction protein assembly, thereby reducing pathogen invasion and colonization (Liu et al. 2021). In this study, the concentrations of acetate, propionate, butyrate, and total SCFA in the cecal content of 21‐day‐old broilers were significantly elevated in the group receiving a 6% FCSM diet compared to the control group. These results were consistent with the research of Gu et al. (2021), who reported that dietary FCSM significantly elevated SCFA concentrations in the ileum and cecum of piglets. Existing evidence suggests that acetate, propionate, and butyrate exhibit anti‐inflammatory properties by downregulating the expression of cytokines such as TNFα, IL‐4, and IL‐5 via receptor activation or histone deacetylase inhibition pathways (Ali et al. 2022). Furthermore, butyrate is known to enhance intestinal barrier function by promoting the assembly of tight junctions (Liu et al. 2021). These mechanisms collectively elucidate the observed improvements in intestinal morphology and immune performance in the 6% FCSM group. In summary, the alteration in SCFA may represent a key mechanism by which FCSM improves immune function and intestinal morphology, thereby enhancing production performance.

## 5 Conclusion

In conclusion, this study conducted a systematic evaluation of the nutritional value of FCSM and its impact on growth performance, immune performance, intestinal morphology, cecal microbiota composition, and SCFA production in broilers. Fermentation effectively improves the nutritional profile and amino acid digestibility (specifically glutamate and valine) of CSM. In broilers, incorporating 6%–9% FCSM into the diet consistently improves systemic immune function and cecal microbial health over a 42‐day period. Specifically, a 6% inclusion of FCSM optimizes intestinal morphology and SCFA production, while a 9% inclusion notably enhances growth performance during the starter phase and serves as a viable functional protein source to replace 30% of SBM in the total diet without compromising overall growth.

## Author Contributions

**Dan Song:** software, formal analysis, data curation, writing – original draft, visualization, validation. **Qiuxi Xuan:** software, data curation, formal analysis, investigation, writing – original draft, visualization. **Beibei He:** methodology, data curation, investigation. **Xiaolin Hou:** conceptualization, writing – review and editing, supervision. **Yongwei Wang:** methodology, resources. **Weiwei Wang:** methodology, resources, supervision, project administration. **Kuanbo Liu:** methodology, validation. **Tao Duan:** validation, investigation. **Junlin Cheng:** investigation. **Aike Li:** conceptualization, writing – review and editing, supervision, project administration, funding acquisition. **Lin Qiao:** conceptualization, methodology, formal analysis, resources, data curation, supervision, writing – review and editing, funding acquisition, project administration.

## Funding

Guangdong Key‐Area R&D Program, Department of Science and Technology of Guangdong Province, People's Republic of China, 2020B0202010001.Development of Fermented Feed Processing Technology Based on Rapeseed Meal and Distillers' Grains, H26002.

## Ethics Statement

The authors have nothing to report.

## Consent

The authors have nothing to report.

## Conflicts of Interest

The authors declare no conflicts of interest.

## Supporting information


**Table S1:** Standardized ileal amino acid digestibility coefficients of experimental diets in broiler chickens (%, air‐dry basis).

## Data Availability

The data presented in this study are available on request from the corresponding author.

## References

[fsn371765-bib-0001] Ali, Q. , S. Ma , S. La , et al. 2022. “Microbial Short‐Chain Fatty Acids: A Bridge Between Dietary Fibers and Poultry Gut Health—A Review.” Animal Bioscience 35, no. 10: 1461–1478. 35507857 10.5713/ab.21.0562 PMC9449382

[fsn371765-bib-0002] Ashayerizadeh, A. , V. Jazi , M. R. Rezvani , H. Mohebodini , E. A. Soumeh , and M. R. Abdollahi . 2024. “An Investigation Into the Influence of Fermented Cottonseed Meal on the Productive Performance, Egg Quality, and Gut Health in Laying Hens.” Poultry Science 103, no. 5: 103574. 10.1016/j.psj.2024.103574 PMC10999706 38564832

[fsn371765-bib-0003] Ashayerizadeh, A. , V. Jazi , F. Sharifi , et al. 2024. “Fermented but Not Irradiated Cottonseed Meal Has the Potential to Partially Substitute Soybean Meal in Broiler Chickens.” Animals (Basel) 14, no. 19: 2797. 39409746 10.3390/ani14192797 PMC11475882

[fsn371765-bib-0004] Esen, S. 2024. “Effect of Solid‐State Fermented Cottonseed Meal on Broiler Growth Performance, Carcass Traits and Blood Biochemical Parameters: A Systematic Review.” Fermentation 10, no. 11: 562.

[fsn371765-bib-0005] FAOSTAT . 2025. “FAO Statistical Database. the Food and Agriculture Organization of the United Nations, Rome.” https://www.fao.org/faostat/zh/#compare.

[fsn371765-bib-0006] Farahat, M. , D. Ibrahim , A. T. Y. Kishawy , H. M. Abdallah , A. Hernandez‐Santana , and G. Attia . 2021. “Effect of Cereal Type and Plant Extract Addition on the Growth Performance, Intestinal Morphology, Caecal Microflora, and Gut Barriers Gene Expression of Broiler Chickens.” Animal 15, no. 3: 100056. 33573933 10.1016/j.animal.2020.100056

[fsn371765-bib-0007] Feng, J. , X. Liu , Z. R. Xu , Y. Z. Wang , and J. X. Liu . 2007. “Effects of Fermented Soybean Meal on Digestive Enzyme Activities and Intestinal Morphology in Broilers.” Poultry Science 86, no. 6: 1149–1154. 10.1093/ps/86.6.1149 17495085

[fsn371765-bib-0008] Fruci, M. , M. Kithama , E. G. Kiarie , et al. 2023. “Effects of Partial or Complete Replacement of Soybean Meal With Commercial Black Soldier Fly Larvae ( *Hermetia illucens* ) Meal on Growth Performance, Cecal Short Chain Fatty Acids, and Excreta Metabolome of Broiler Chickens.” Poultry Science 102: 102463. 10.1016/j.psj.2022.102463 PMC9941379 36758368

[fsn371765-bib-0009] Ghiasvand, A. R. , A. Khatibjoo , Y. Mohammadi , M. Akbari Gharaei , and H. Shirzadi . 2021. “Effect of Fennel Essential Oil on Performance, Serum Biochemistry, Immunity, Ileum Morphology and Microbial Population, and Meat Quality of Broiler Chickens Fed Corn or Wheat‐Based Diet.” British Poultry Science 62, no. 4: 562–572. 10.1080/00071668.2021.1883551 33530744

[fsn371765-bib-0010] Gong, H. , F. Liang , C. Cai , et al. 2025. “Dietary *Saccharomyces cerevisiae* Fermentation Product Improved Egg Quality by Modulating Intestinal Health, Ovarian Function, and Cecal Microbiota in Post‐Peak Laying Hens.” Poultry Science 104, no. 5: 104979. 10.1016/j.psj.2025.104979 PMC11950754 40073632

[fsn371765-bib-0011] Gu, X. , Z. Li , J. Wang , et al. 2021. “Fermented Cottonseed Meal as a Partial Replacement for Soybean Meal Could Improve the Growth Performance, Immunity and Antioxidant Properties, and Nutrient Digestibility by Altering the Gut Microbiota Profile of Weaned Piglets.” Frontiers in Microbiology 12: 734389. 34539619 10.3389/fmicb.2021.734389 PMC8440953

[fsn371765-bib-0012] Han, F. , J. Qian , Y. Qu , et al. 2022. “Partial Replacement of Soybean Meal With Fermented Cottonseed Meal in a Low Fishmeal Diet Improves the Growth, Digestion and Intestinal Microbiota of Juvenile White Shrimp *Litopenaeus vannamei* .” Aquaculture Reports 27: 101339.

[fsn371765-bib-0014] Kasaeizadeh, R. , S. Salari , M. R. Abdollahi , and F. Baghban . 2025. “Changes in Performance, Apparent Ileal Digestibility and Intestinal Morphology of Broiler Chickens Fed Diets Containing Sunflower Hulls With Full‐Fat Canola Seed.” Animal Feed Science and Technology 327: 116417.

[fsn371765-bib-0015] Li, J. , T. Gao , Z. Hao , X. Guo , and B. Zhu . 2022. “Anaerobic Solid‐State Fermentation With *Bacillus subtilis* for Digesting Free Gossypol and Improving Nutritional Quality in Cottonseed Meal.” Frontiers in Nutrition 9: 1017637. 36570163 10.3389/fnut.2022.1017637 PMC9773203

[fsn371765-bib-0016] Li, X. , H. Ling , Z. He , et al. 2024. “Effects of Edible Grass ( *rumex patientia* l. x Rumex Tianschanicus a. los) Leaf Powder on Growth Performance, Antioxidant Properties, Cecal Short‐Chain Fatty Acids, and Microbial Community Levels in Broilers.” Antioxidants (Basel) 13, no. 11: 1291. 39594435 10.3390/antiox13111291 PMC11590973

[fsn371765-bib-0017] Liu, B. , H. Liu , D. Liu , et al. 2024. “Free Gossypol Removal and Nutritional Value Enhancement of Cottonseed Meal via Solid‐State Fermentation With *rhodotorula mucilaginosa* TG529.” Agriculture 14, no. 9: 1463.

[fsn371765-bib-0018] Liu, J. , E. Puolanne , M. Schwartzkopf , and A. Arner . 2020. “Altered Sarcomeric Structure and Function in Woody Breast Myopathy of Avian Pectoralis Major Muscle.” Frontiers in Physiology 11: 287. 32328000 10.3389/fphys.2020.00287 PMC7160512

[fsn371765-bib-0019] Liu, L. , Q. Li , Y. Yang , and A. Guo . 2021. “Biological Function of Short‐Chain Fatty Acids and Its Regulation on Intestinal Health of Poultry.” Frontiers in Veterinary Science 8: 736739. 34733901 10.3389/fvets.2021.736739 PMC8558227

[fsn371765-bib-0020] Moreno‐Mendoza, Y. , K. D. Lopez‐Villarreal , C. A. Hernandez‐Martinez , et al. 2021. “Effect of Moringa Leaf Powder and Agave Inulin on Performance, Intestinal Morphology, and Meat Yield of Broiler Chickens.” Poultry Science 100, no. 2: 738–745. 10.1016/j.psj.2020.11.058 PMC7858189 33518127

[fsn371765-bib-0021] Munawar, Z. , S. Amjid , F. Ramzan , et al. 2025. “Effects of Partial Soybean Meal Replacement With Sunflower Meal and Non‐Starch Polysaccharide Degrading Enzymes Supplementation on Broiler Growth Performance, Nutrient Digestibility, and Gut Morphology.” Veterinary World 18, no. 3: 695–704. 40342752 10.14202/vetworld.2025.695-704 PMC12056904

[fsn371765-bib-0022] Niu, J. L. , L. Q. Wei , Y. Q. Luo , et al. 2021. “Fermented Cottonseed Meal Improves Production Performance and Reduces Fat Deposition in Broiler Chickens.” Animal Bioscience 34, no. 4: 680–691. 33254361 10.5713/ajas.20.0571 PMC7961297

[fsn371765-bib-0023] NRC . 1994. Nutrient Requirements of Poultry in Book Nutrient Requirements of Poultry. National Academy Press.

[fsn371765-bib-0024] Rehemujiang, H. , H. A. Yusuf , T. Ma , et al. 2023. “Fermented Cottonseed and Rapeseed Meals Outperform Soybean Meal in Improving Performance, Rumen Fermentation, and Bacterial Composition in Hu Sheep.” Frontiers in Microbiology 14: 1119887. 37007511 10.3389/fmicb.2023.1119887 PMC10060860

[fsn371765-bib-0025] Sakwiwatkul, K. , W. Ojan , P. Treehera , et al. 2025. “Impact of Yam Bean Pulp on Growth Performance, Gut Morphology, Digestive Development, and Digestibility in Broilers Raised in Hot Environments.” Animal Bioscience 38, no. 10: 2203–2214. 40400193 10.5713/ab.25.0057 PMC12415378

[fsn371765-bib-0039] Song, D. , T. Duan , R. Li , et al. 2025. “Multi‐Omics Insights Into the Role of Fructooligosaccharides Supplementation in Alleviating Salpingitis in Laying Hens.” Animal Research & One Health 4, no. 1: 105–119.

[fsn371765-bib-0026] Song, D. , A. Li , Y. Wang , et al. 2022. “Effects of Synbiotic on Growth, Digestibility, Immune and Antioxidant Performance in Broilers.” Animal 16, no. 4: 100497. 35338905 10.1016/j.animal.2022.100497

[fsn371765-bib-0027] Song, D. , Y. W. Wang , Y. J. Hou , Z. L. Dong , W. W. Wang , and A. K. Li . 2016. “The Effects of Dietary Supplementation of Microencapsulated *Enterococcus Faecalis* and the Extract of Camellia Oleifera Seed on Growth Performance, Immune Functions, and Serum Biochemical Parameters in Broiler Chickens.” Journal of Animal Science 94, no. 8: 3271–3277. 27695776 10.2527/jas.2016-0286

[fsn371765-bib-0028] Soumeh, E. A. , H. Mohebodini , M. Toghyani , A. Shabani , A. Ashayerizadeh , and V. Jazi . 2019. “Synergistic Effects of Fermented Soybean Meal and Mannan‐Oligosaccharide on Growth Performance, Digestive Functions, and Hepatic Gene Expression in Broiler Chickens.” Poultry Science 98, no. 12: 6797–6807. 10.3382/ps/pez409 PMC8913979 31347672

[fsn371765-bib-0029] Teng, P. Y. , S. Yadav , H. Shi , and W. K. Kim . 2021. “Evaluating Endogenous Loss and Standard Ileal Digestibility of Amino Acids in Response to the Graded Severity Levels of *E. maxima* Infection.” Poultry Science 100, no. 11: 101426. 10.1016/j.psj.2021.101426 PMC8463777 34547620

[fsn371765-bib-0030] Wang, Y. , Q. Deng , D. Song , et al. 2017. “Effects of Fermented Cottonseed Meal on Growth Performance, Serum Biochemical Parameters, Immune Functions, Antioxidative Abilities, and Cecal Microflora in Broilers.” Food and Agricultural Immunology 28, no. 4: 725–738.

[fsn371765-bib-0031] Weimer, S. L. , S. Zuelly , M. Davis , D. M. Karcher , and M. A. Erasmus . 2022. “Differences in Carcass Composition and Meat Quality of Conventional and Slow‐Growing Broiler Chickens Raised at 2 Stocking Densities.” Poultry Science 101, no. 6: 101833. 10.1016/j.psj.2022.101833 PMC9018444 35421814

[fsn371765-bib-0032] Wu, Z. , J. Liu , J. Chen , et al. 2020. “Effects of Fermentation on Standardized Ileal Digestibility of Amino Acids and Apparent Metabolizable Energy in Rapeseed Meal Fed to Broiler Chickens.” Animals (Basel) 10, no. 10: 1774. 33019513 10.3390/ani10101774 PMC7599665

[fsn371765-bib-0033] Xie, S. S. , J. J. Shen , Y. Liu , et al. 2024. “Effects of Fermented Cottonseed Meal Inclusions on Growth Performance, Serum Biochemical Parameters and Hepatic Lipid Metabolism of Geese During 28‐70 d of Age.” Poultry Science 103, no. 6: 103702. 10.1016/j.psj.2024.103702 PMC11063510 38652950

[fsn371765-bib-0034] Xuan, Q. , L. Qiao , X. Hou , et al. 2022. “Screening of Strains for Solid‐State Fermentation of Raw Cottonseed Meal and Study on Fermentation Technology.” Chinese Journal of Animal Nutrition 34, no. 5: 3376–3391.

[fsn371765-bib-0035] Yan, L. , S. An , X. Lv , et al. 2025. “Effects of Replacing Soybean Meal With Cottonseed Meal on Growth Performance, Carcass Trait, Intestinal Development and Intestinal Microbiota of Broiler Chickens.” Poultry Science 104, no. 2: 104653. 10.1016/j.psj.2024.104653 PMC11786066 39823832

[fsn371765-bib-0036] Yao, T. , C. Wang , L. Liang , et al. 2024. “Effects of Fermented Sweet Potato Residue on Nutrient Digestibility, Meat Quality, and Intestinal Microbes in Broilers.” Animal Nutrition 17: 75–86. 38737580 10.1016/j.aninu.2024.03.007 PMC11087712

[fsn371765-bib-0037] Zhu, F. , B. Zhang , J. Li , and L. Zhu . 2020. “Effects of Fermented Feed on Growth Performance, Immune Response, and Antioxidant Capacity in Laying Hen Chicks and the Underlying Molecular Mechanism Involving Nuclear Factor‐kappaB.” Poultry Science 99, no. 5: 2573–2580. 10.1016/j.psj.2019.12.044 PMC7597451 32359593

[fsn371765-bib-0038] Zhuo, Y. , X. Zou , Y. Wang , et al. 2023. “Standardized Ileal Digestibility of Amino Acids in Cottonseed Meal Fed to Pregnant and Non‐Pregnant Sows.” Journal of Animal Science 101: 1–11. 10.1093/jas/skad132 PMC10199789 37119089

