# Supplementary material for: Optimizing Broiler Diets: Nutritional and Functional Benefits of Fermented Cottonseed Meal
Source: Food Sci Nutr. 2026 Jul 3;14(7):e71765. doi: 10.1002/fsn3.71765 (PMC13332134; doi:10.1002/fsn3.71765)
Supplement: Supplementary file 1 — Table S1: Standardized ileal amino acid digestibility coefficients of experimental diets in broiler chickens (%, air‐dry basis). [file FSN3-14-e71765-s001.docx]

Table S1 Standardized ileal amino acid digestibility coefficients of experimental diets in broiler chickens (%, air-dry basis)

| Items | CSM | FCSM | NFD |
| --- | --- | --- | --- |
| CSM | 50.00 | 0.00 | 0.00 |
| FCSM | 0.00 | 48.50 | 0.00 |
| Glucose | 42.05 | 43.55 | 19.98 |
| Soybean oil | 4.00 | 4.00 | 3.00 |
| Dicalcium phosphate | 1.60 | 1.60 | 0.00 |
| Calcium hydrogen phosphate anhydrous | 0.00 | 0.00 | 1.80 |
| Limestone | 1.20 | 1.20 | 1.20 |
| Sodium chloride | 0.30 | 0.30 | 0.35 |
| Choline chloride | 0.10 | 0.10 | 0.10 |
| Premix ^1^ | 0.25 | 0.25 | 0.25 |
| Titanium dioxide | 0.50 | 0.50 | 0.50 |
| Corn starch | 0.00 | 0.00 | 67.82 |
| Microcrystalline cellulose | 0.00 | 0.00 | 5.00 |
| Total, % | 100.00 | 100.00 | 100.00 |
| Nutritional levels^2^, % | | | |
| ME（kcal/kg） | 2645 | 2578 | 3009 |
| Crude protein | 22.09 | 22.04 | 0.26 |
| Calcium | 0.94 | 0.94 | 0.96 |
| Total phosphorus | 0.81 | 0.79 | 0.41 |
| Lysine | 0.99 | 1.03 | 0.00 |
| Methionine | 0.29 | 0.32 | 0.00 |

^1^ The premix provided the following per kg of diets: vitamin A, 9,500 IU; vitamin D_3_, 62.5 ug; vitamin K_3_, 2.65mg; vitamin B_12_, 0.025 mg; vitamin B_2_, 6 mg; vitamin E, 30 IU; biotin, 0.0325 mg; folic acid,1.25 mg; pantothenic acid 12 mg; nicotinic acid, 50 mg; Cu, 8 mg; Zn, 75 mg; Fe, 80 mg; Mn,100 mg; Se, 0.15 mg; I, 0.35 mg.

^2^Nutrient content were calculated values.

CSM: Conttonseed meal; FCSM: Fermented cottonseed meal; NFD: nitrogen-free diet; ME: Metabolizable energy.
